# Draft Genome Sequence of Bacillus velezensis Strain ZeaDK315Endo16

**DOI:** 10.1128/MRA.00136-19

**Published:** 2019-11-14

**Authors:** Bukola Rhoda Aremu, Claire Prigent-Combaret, Olubukola Oluranti Babalola

**Affiliations:** a North-West University, Food Security and Safety Niche Area, Faculty of Natural and Agricultural Sciences, Mmabatho, South Africa; b University of Lyon, The Rhizosphere Team, UMR CNRS 5557 Microbial Ecology, Villeurbanne, France; Indiana University, Bloomington

## Abstract

Here, we report the draft genome sequence of the endophytic Bacillus velezensis strain ZeaDK315Endo16, isolated from DK315 maize from Lyon, France. *B. velezensis* ZeaDK315Endo16 exhibits a suppressive ability toward Fusarium graminearum, a widely known threat to maize production and quality.

## ANNOUNCEMENT

**FIG 1:**
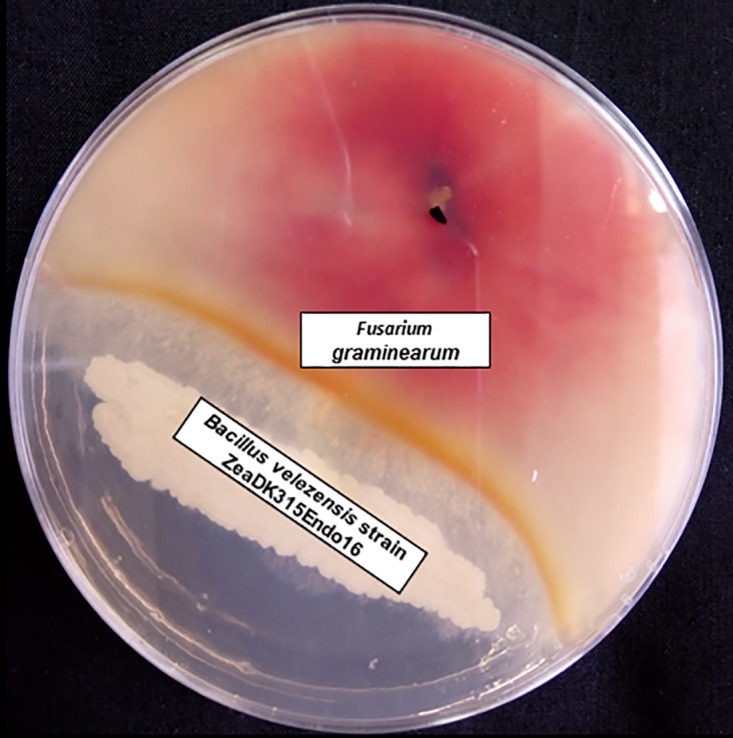
Inhibition of Fusarium graminearum by a single loopful of Bacillus velezensis strain ZeaDK315Endo16 antagonist streaked 3 days after the fungi was inoculated on the opposite sides.

Globally, there is a setback in maize productivity and quality because of Fusarium graminearum infestation, leading to great economic losses (1). F. graminearum not only contaminates maize but also impairs the health of animals and humans upon consumption of maize. To circumvent the challenge by this fungus, chemical control measures have been implemented which are expensive and increase environmental pollution (2). Hence, the need arises for a safer and environmentally friendly control measure like the use of a bioinoculum as a means of biocontrol (3). Thus, the endophytic bacteria were isolated from the maize seed, examined *in vitro* on potato dextrose agar (PDA) contained in petri dishes for their ability to suppress F. graminearum, and gave a notable inhibition zone (Fig. 1). Bacillus velezensis strain ZeaDK315Endo16 elicited the most significant inhibitory factor against this pathogen. The endophytic plant-microbe interaction needs to be investigated to gain insight into the genomic composition of endophytic *B. velezensis* strain ZeaDK315Endo16 and recognition of the gene responsible for this suppressive ability. We isolated *B. velezensis* ZeaDK315Endo16 from the seed of DK315 maize obtained from Lyon, France. *B. velezensis* ZeaDK315Endo16 was cultivated on Luria-Bertani agar (Sigma-Aldrich Corporation, USA) in a petri dish and was incubated at 25°C for 24 h. The ZR fungal/bacterial DNA kit (Zymo Research Corp., Irvine, CA, USA) was utilized for genomic DNA extraction of the pure culture. A draft genome of *B. velezensis* strain ZeaDK315Endo16 was obtained by direct sequencing of genomic DNA using the Illumina HiSeq 2500 system (rapid mode) at MR DNA (Shallowater, TX, USA). The KAPA HyperPlus kit (Roche) was used in library preparation to generate 250-bp Illumina paired-end reads. FastQC v.1.0.4 was utilized for sequence quality (Babraham Bioinformatics, United Kingdom); the low-quality reads of <30 and adapters were removed with PRINSEQ v.0.0.4 (4) and Cutadapt v.1.0.7 (5). The high-quality edited sequence reads were *de novo* assembled with SPAdes v.3.12.0 (6), resulting in a total genome assembly size of 4,452,329 bp with 788 scaffolds. The quality of the genome assemblies was checked using QUAST v.5.0.0 (7). Reassembly with Filter Assembled Contigs by Length v.1.1.2 in the Kbase platform (8) removed the contigs with unusual G+C content and sequencing depth that likely result from contamination. Finally, a genome with a size of 3,925,184 bp with 1 contig, a G+C content of 46.4%, an *N*_50_ value of 3,925,184 bp, and an *L*_50_ of 1 was obtained. The NCBI Prokaryotic Genome Annotation Pipeline (PGAP) v.4.7 was utilized for the genome annotation (9). The genome in total contains 3,930 genes, 3,843 coding DNA sequences (CDSs), 3,747 CDSs (with protein), 87 genes (RNA), 9 rRNAs, 73 tRNAs, 5 noncoding RNAs (ncRNAs), and 96 pseudogenes. There is only one questionable CRISPR in the genome detected by CRISPRFinder (10). Additionally, the major secondary metabolites for the suppressiveness of F. graminearum are macrolactin, bacillaene, fengycin, difficidin, bacillibactin, bacilysin, mersacidin, and surfactin, identified using antiSMASH v.5.0.0 (11). This draft genome sequence may serve as an inoculum for biocontrol. Analysis (average nucleotide identity [ANI]) of the genome of *B. velezensis* ZeaDK315Endo16 shows a high similarity with *B. velezensis* (identity, 98.5%). Default parameters were used for all software.

### Data availability.

This whole-genome shotgun project has been deposited at DDBJ/ENA/GenBank under the accession number CP043809. The version described in this paper is the first version, SDSJ00000000.1. The raw genome sequence data are under the SRA study accession number SRP181915.
